# Supplementary material for: Drug Discovery Using Evolutionary Similarities in Chemical Binding to Inhibit Patient-Derived Hepatocellular Carcinoma
Source: Int J Mol Sci. 2022 Jul 19;23(14):7971. doi: 10.3390/ijms23147971 (PMC9322808; doi:10.3390/ijms23147971)
Supplement: Supplementary file 1 [file ijms-23-07971-s001.zip › Supplementary Figure S1.pptx]

## Slide 1
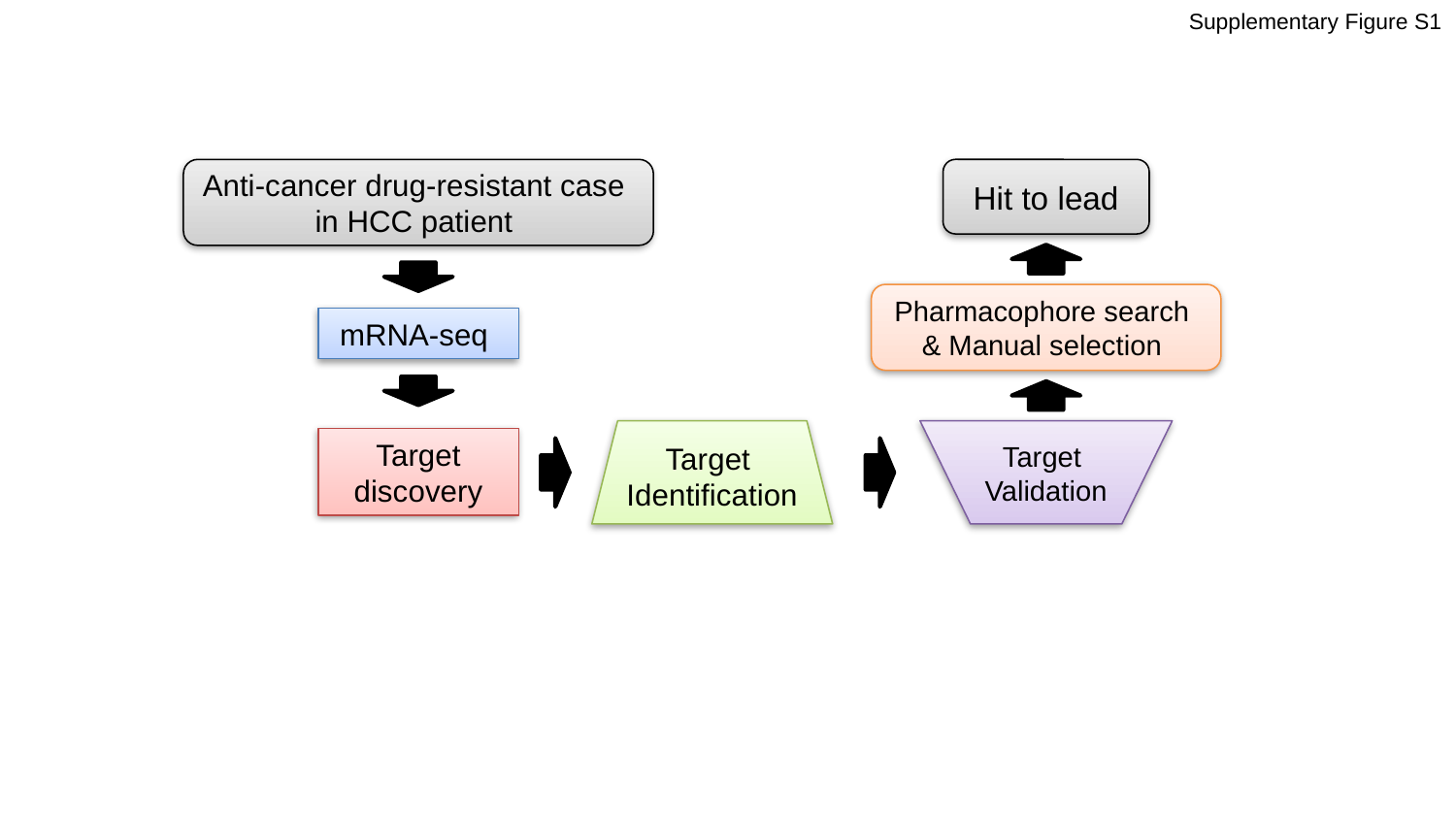

Supplementary Figure S1
Anti-cancer drug-resistant case
in HCC patient
Hit to lead
Pharmacophore search
& Manual selection
mRNA-seq
Target
Identification
Target
Validation
Target discovery
