# Supplementary material for: Drug Discovery Using Evolutionary Similarities in Chemical Binding to Inhibit Patient-Derived Hepatocellular Carcinoma
Source: Int J Mol Sci. 2022 Jul 19;23(14):7971. doi: 10.3390/ijms23147971 (PMC9322808; doi:10.3390/ijms23147971)
Supplement: Supplementary file 1 [file ijms-23-07971-s001.zip › Supplementary image, S4.pdf]

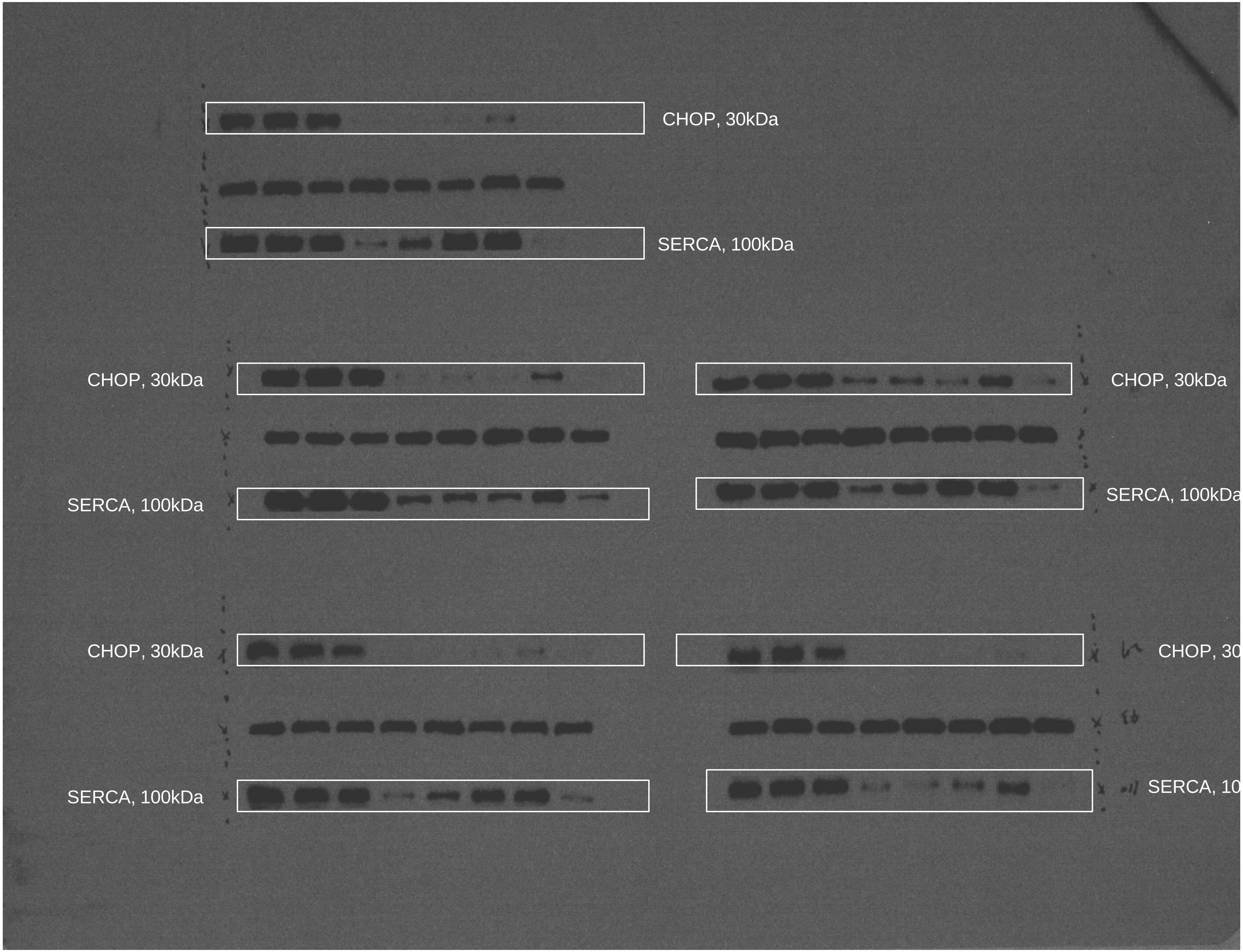
CHOP, 30kDa
SERCA, 100kDa
CHOP, 30kDa
CHOP, 30kDa
SERCA, 100kDa
SERCA, 100kDa
CHOP, 30kDa
CHOP, 30
SERCA, 100kDa
SERCA, 10

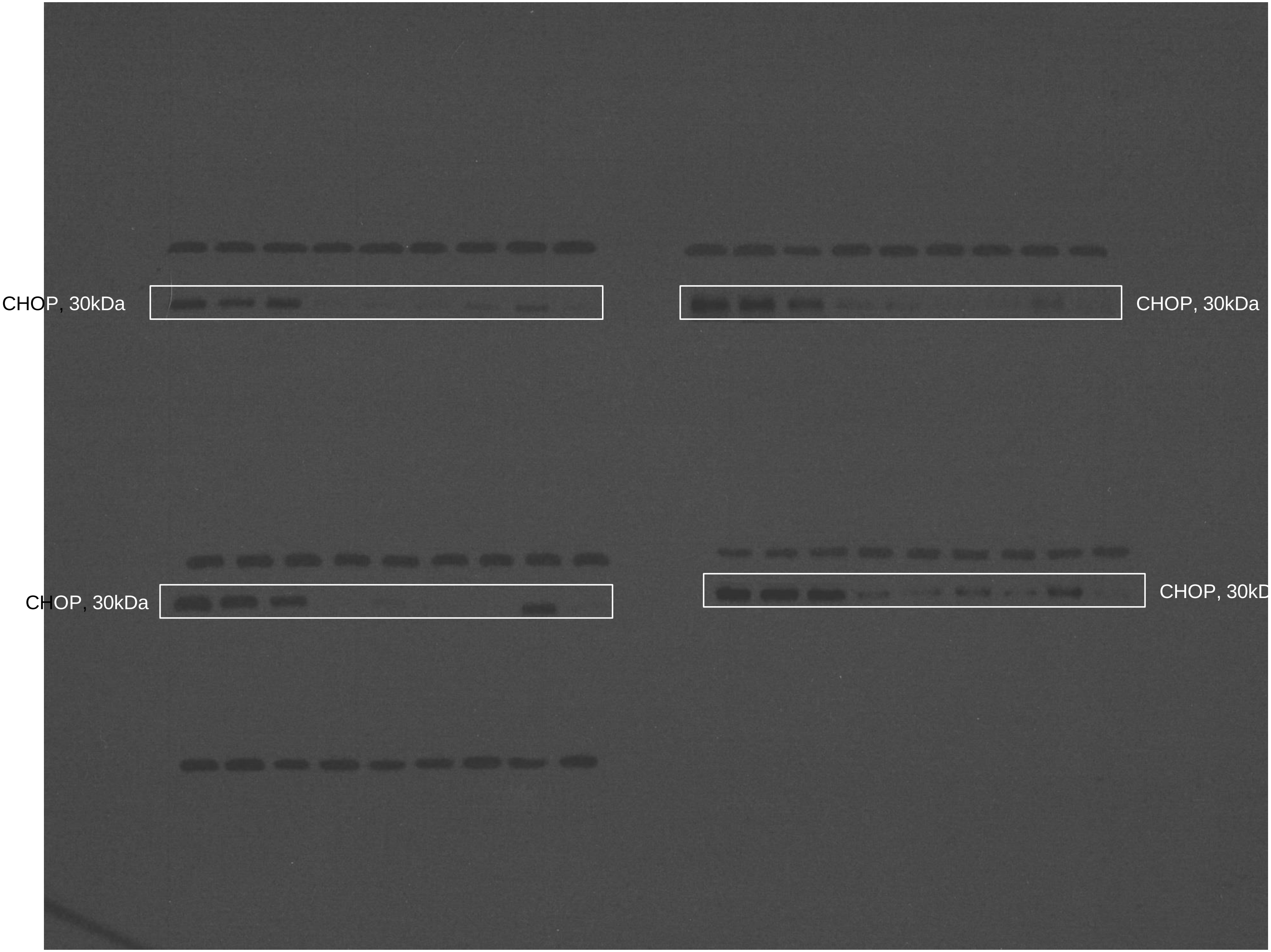
CHOP, 30kDa
CHOP, 30kDa
CHOP, 30kDa
CHOP, 30kD

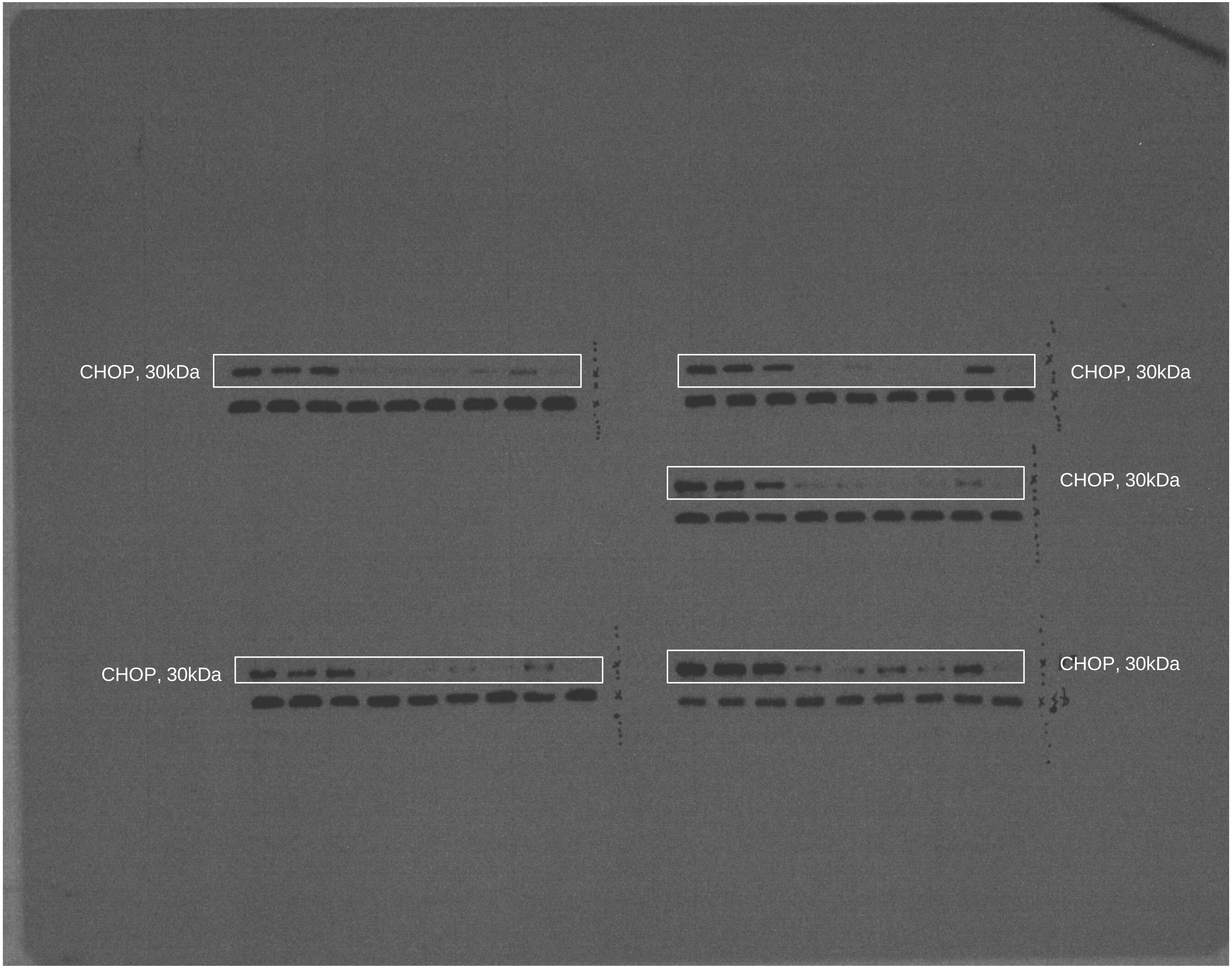
CHOP, 30kDa
CHOP, 30kDa
CHOP, 30kDa
CHOP, 30kDa
CHOP, 30kDa
