# Supplementary material for: Drug Discovery Using Evolutionary Similarities in Chemical Binding to Inhibit Patient-Derived Hepatocellular Carcinoma
Source: Int J Mol Sci. 2022 Jul 19;23(14):7971. doi: 10.3390/ijms23147971 (PMC9322808; doi:10.3390/ijms23147971)
Supplement: Supplementary file 1 [file ijms-23-07971-s001.zip › Supplementary Table S1.pdf]

Supplementary Table S1. Primer sequences for qRT-PCR.

Gene	Forward primer (5'-3')	Reverse primer (5'-3')
SERCA1	GTGATCCGCCAGCTAATG	CGAATGTCAGGTCCGTCT
SERCA2	GGTGGTTCATTGCTGCTGAC	TTTCGGACAAGCTGTTGAGG
SERCA3	GATGGAGTGAACGACGCA	CCAGGTATCGGAAGAAGAG
α-tubulin	CGGGCAGTGTTTGTAGACTTGG	CTCCTTGCCAATGGTGTAGTGC
