# Supplementary figures and images for: Drug Discovery Using Evolutionary Similarities in Chemical Binding to Inhibit Patient-Derived Hepatocellular Carcinoma
Source: Int J Mol Sci. 2022 Jul 19;23(14):7971. doi: 10.3390/ijms23147971 (PMC9322808; doi:10.3390/ijms23147971)

## Slide 1
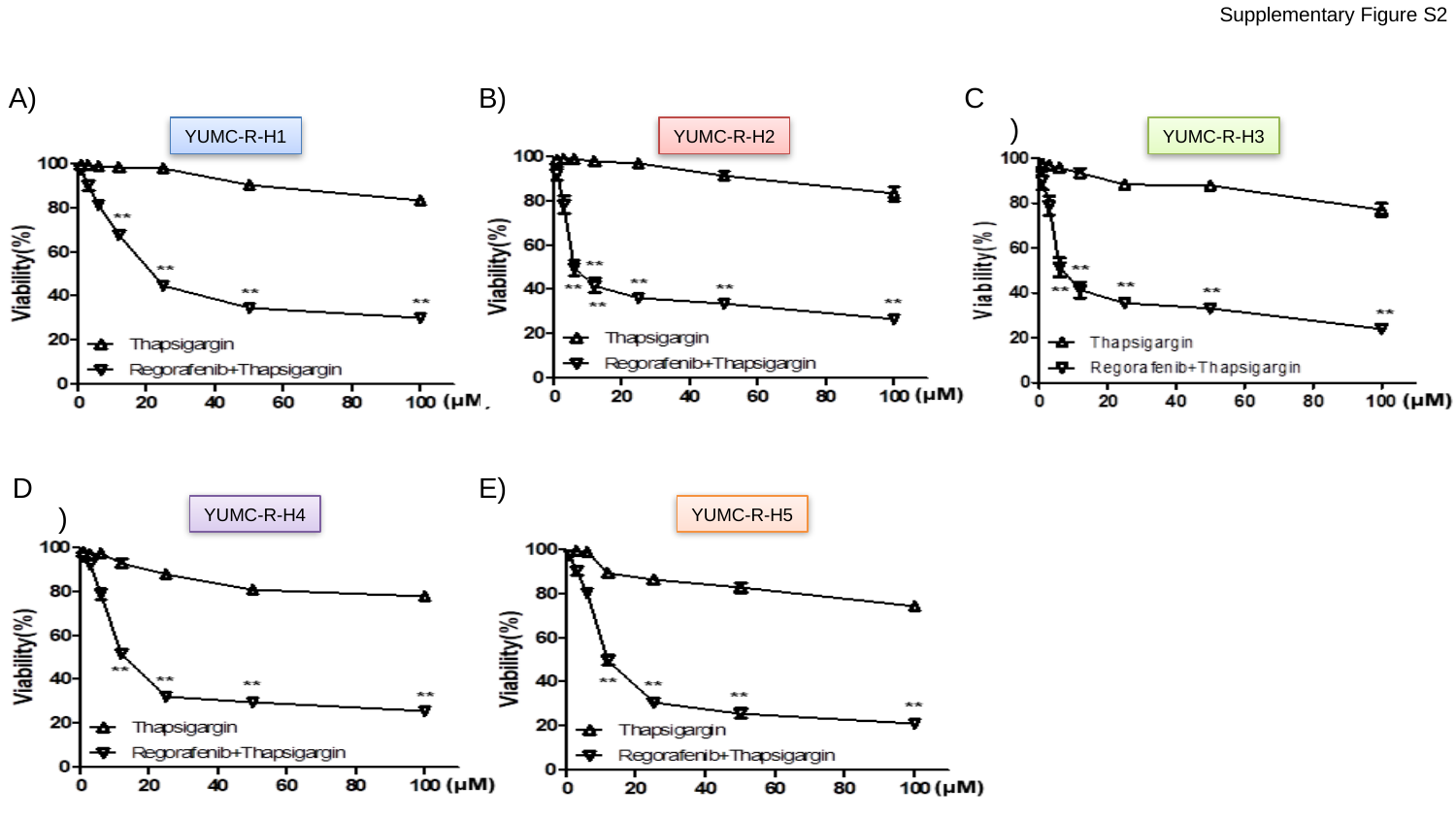

Supplementary Figure S2
A)
B)
C)
YUMC-R-H1
YUMC-R-H2
YUMC-R-H3
D)
E)
YUMC-R-H4
YUMC-R-H5

Supplement: Supplementary file 1 [file ijms-23-07971-s001.zip › Supplementary Figure S2.pptx]

## Slide 1
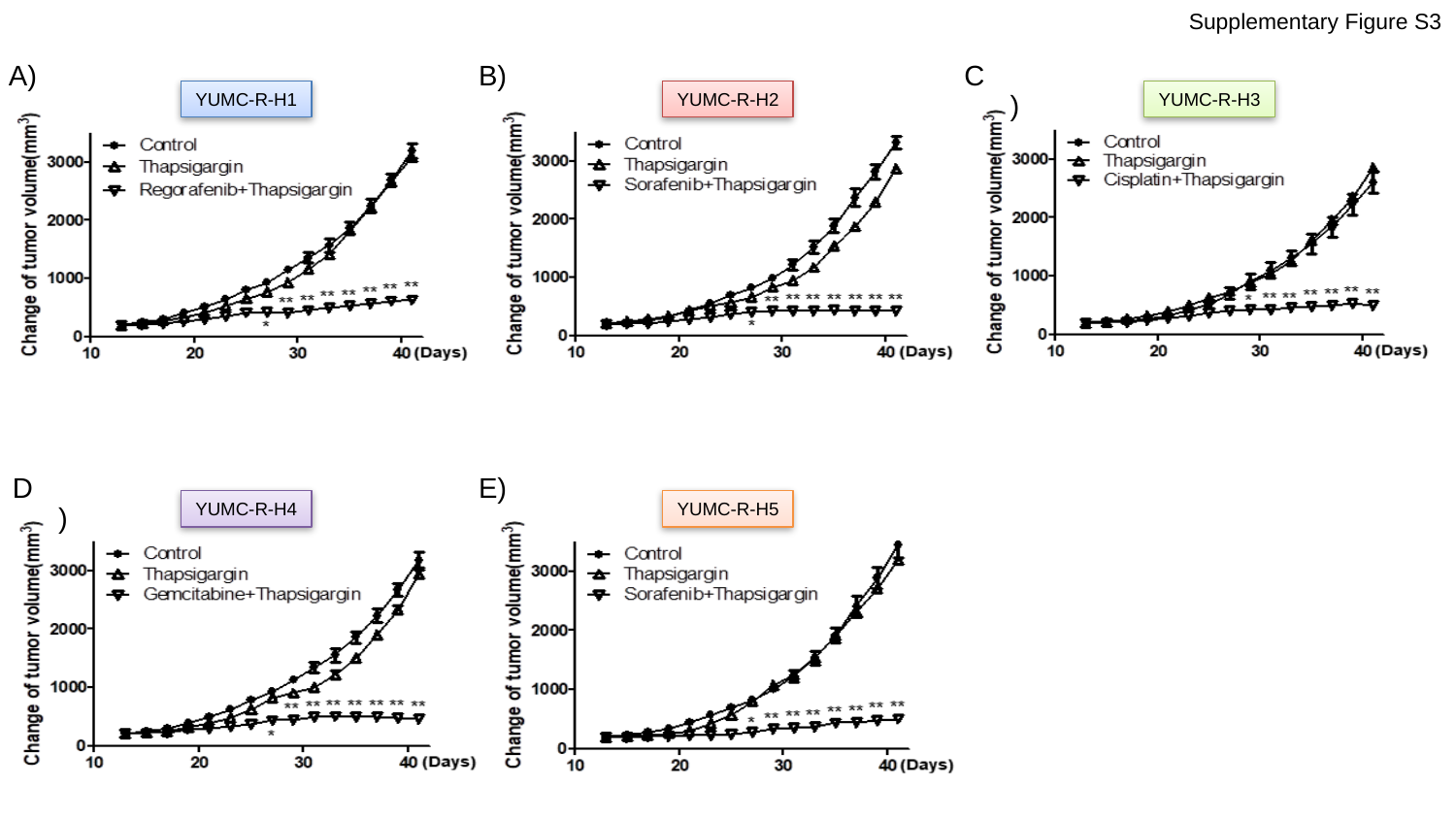

Supplementary Figure S3
A)
B)
C)
YUMC-R-H1
YUMC-R-H2
YUMC-R-H3
D)
E)
YUMC-R-H4
YUMC-R-H5

Supplement: Supplementary file 1 [file ijms-23-07971-s001.zip › Supplementary Figure S3.pptx]
